# Low Frequency Variants in the Exons Only Encoding Isoform A of *HNF1A* Do Not Contribute to Susceptibility to Type 2 Diabetes

**DOI:** 10.1371/journal.pone.0006615

**Published:** 2009-08-12

**Authors:** Bahram Jafar-Mohammadi, Christopher J. Groves, Katharine R. Owen, Timothy M. Frayling, Andrew T. Hattersley, Mark I. McCarthy, Anna L. Gloyn

**Affiliations:** 1 Oxford Centre for Diabetes, Endocrinology and Metabolism, University of Oxford, Oxford, United Kingdom; 2 Oxford Biomedical Research Centre, Churchill Hospital, Oxford, United Kingdom; 3 Institute of Biomedical and Clinical Science, Peninsula Medical School, Exeter, United Kingdom; 4 Wellcome Trust Centre for Human Genetics, University of Oxford, Oxford, United Kingdom; Institute of Preventive Medicine, Denmark

## Abstract

**Background:**

There is considerable interest in the hypothesis that low frequency, intermediate penetrance variants contribute to the proportion of Type 2 Diabetes (T2D) susceptibility not attributable to the common variants uncovered through genome-wide association approaches. Genes previously implicated in monogenic and multifactorial forms of diabetes are obvious candidates in this respect. In this study, we focussed on exons 8–10 of the *HNF1A* gene since rare, penetrant mutations in these exons (which are only transcribed in selected *HNF1A* isoforms) are associated with a later age of diagnosis of Maturity onset diabetes of the young (MODY) than mutations in exons 1–7. The age of diagnosis in the subgroup of HNF1A-MODY individuals with exon 8–10 mutations overlaps with that of early multifactorial T2D, and we set out to test the hypothesis that these exons might also harbour low-frequency coding variants of intermediate penetrance that contribute to risk of multifactorial T2D.

**Methodology and Principal Findings:**

We performed targeted capillary resequencing of *HNF1A* exons 8–10 in 591 European T2D subjects enriched for genetic aetiology on the basis of an early age of diagnosis (≤45 years) and/or family history of T2D (≥1 affected sibling). PCR products were sequenced and compared to the published *HNF1A* sequence. We identified several variants (rs735396 [IVS9−24T>C], rs1169304 [IVS8+29T>C], c.1768+44C>T [IVS9+44C>T] and rs61953349 [c.1545G>A, p.T515T] but no novel non-synonymous coding variants were detected.

**Conclusions and Significance:**

We conclude that low frequency, nonsynonymous coding variants in the terminal exons of *HNF1A* are unlikely to contribute to T2D-susceptibility in European samples. Nevertheless, the rationale for seeking low-frequency causal variants in genes known to contain rare, penetrant mutations remains strong and should motivate efforts to screen other genes in a similar fashion.

## Introduction

*HNF1A* encodes the transcription factor hepatocyte nuclear factor 1 alpha and is the gene most commonly implicated in the pathogenesis of symptomatic Maturity-onset diabetes of the young (MODY) [1]. *HNF1A* encodes 3 different isoforms, termed A (encoded by exons 1–10), B (encoded by exons 1–7) and C (encoded by exons 1–6) which arise by alternative splicing and polyadenylation [2]. Two recent reports have demonstrated that mutations in exons 8–10 (which are present only in isoform A) are associated with a later onset of MODY than is the case for mutations in exons 1–7 (18 vs. 25 years, p = 0.0001 and 19 vs. 24 years, p = 0.03) [3], [4]. This difference in presentation is likely to reflect the different temporal and spatial expression profiles of the isoforms (isoform A predominates in the foetal pancreas, adult liver and kidney whilst isoforms B and C predominate in the adult pancreas). Thus, in contrast with mutations which affect the function of all isoforms, mutations in exons 8–10 may display a lower penetrance and later age of onset leading to more clinical overlap with common forms of Type 2 diabetes mellitus (T2D).

Despite the great success of the genome wide association (GWA) approach in identifying common genetic variants which predispose to T2D, the familial aggregation seen in this condition is far from fully explained [5]. The sibling relative risk (λs) for T2D attributable to all currently-known susceptibility variants combined is only ∼1.07, well below the equivalent estimate from epidemiological studies of ∼3.0 [5]. Ongoing efforts to account for this “heritability gap” are increasingly aimed at identification of low frequency (that is, minor allele frequency [MAF] <5%), intermediate penetrance (allelic odds ratios, 2–5) variants. Variants with such characteristics are likely to have remained hidden from view so far, since their frequency is below that targeted by GWA approaches, and the penetrance is insufficient for detection by traditional linkage analyses[6]. Variants with these characteristics have recently been recognised to contribute to susceptibility of other complex traits such as Crohn's disease [7].

Genes already known to play a role in the pathogenesis of monogenic or multifactorial diabetes are logical candidates in which to initiate the search for low frequency causal variants in T2D. In the case of *HNF1A* for example, there is evidence that a private common variant (G319S, located in exon 4) is a major contributor to T2D pathogenesis in the Oji-Cree population (in whom the MAF is 8.7%) [8], [9]. Further evidence that *HNF1A* variants across the allele frequency spectrum play a role in T2D-susceptibility derives from evidence that common variants in this gene are associated with multifactorial T2D [10]–[12].

In the light of this evidence, we reasoned that the terminal isoform-specific exons of *HNF1A* were particularly auspicious candidates in terms of harbouring low frequency, medium-penetrance variants involved in multifactorial diabetes. We set out to test this hypothesis by performing deep resequencing of these exons in a large sample of cases likely to be enriched for such variants, namely those with a strong family history of T2D and/or an early age at diagnosis.

## Materials and Methods

### 

#### Ethics Statement

All subjects gave written informed consent and all protocols were approved by the local ethics committees. The Warren 2 Sibpair collection was approved by multiple local ethics committees in the UK including St Mary's Local Ethics Committee (EC3231). The Young Diabetes in Oxford Cohort was approved by the Oxfordshire Research Ethics Committee A (04/Q1604/97).

To maximize the likelihood of identifying medium penetrance genetic variants influencing T2D-risk, a total of 591 individuals of British ancestry, ascertained for early-adult onset (sample 1, n = 84), and/or family history of T2D (at least 1 sibling affected, sample 2, n = 507) were included (Table 1).

**Table 1:** Characteristics of subjects with T2D.

	Sample 1	Sample 2
**N**	84	507
**Male (%)**	61.9	53.6
**Mean age at diagnosis (years)±SD**	34.3±11.4	55.4±8.5
* **Treatment (ins/OHA/diet) (%)**	26/66/8	14/69/17
**First-degree relative with Diabetes (%)**	48	100
**Mean BMI (kg/m^2^)**±**SD**	32.3±4.8	28.8±5.2

* Treatment at the time of ascertainment. ins, insulin; OHA, oral hypoglycaemic agent.

Sample 1 (the “Young Diabetes in Oxford Cohort”) includes subjects diagnosed with T2D between 18–45 years of age, recruited from Oxfordshire GP practices. We excluded those with type 1 diabetes (T1D) by requiring that all subjects had no permanent requirement for insulin therapy within 12 months of diagnosis and no evidence of islet autoimmunity (glutamic acid decarboxylase (GAD) antibody levels <14 WHO units/ml) [13]. The subjects classified as having T2D did not meet current clinical criteria for MODY diagnostic testing [14] and all had demonstrable fasting C-peptide levels (mean 0.83±0.44 nmols/L [non-diabetic reference range: 0.11–0.61 nmols/L]).

Sample 2 consists of probands from the Warren 2 sibpair collection (W2SP): these have been described previously [15]. All individuals in this group were diagnosed between 35 and 75 years (mean age of diagnosis 55.4 years) and had at least one affected sibling diagnosed with diabetes. Those with positive GAD antibodies were excluded.

To establish the background allele frequency of variants identified in this study in a UK control population, we utilised a subset of the British Birth Cohort of 1958 (n = 350 for the intronic variants identified, n = 1050 for the exonic variant identified) [16], [17].

Targeted capillary resequencing of exons 8–10 of *HNF1A* (including intron-exon boundaries) was performed on the ABI3700 platform (Applied Biosystems, Warrington, UK) using standard protocols (primer sequences are available on request). Sequences were compared to the reference sequence (NM_000545.3) using the unidirectional analysis mode of Mutation surveyor V3.2 (Biogene, Cambridge, UK). This software package has been shown to have a sensitivity of >99% in the unidirectional analysis mode[18]. We further checked the accuracy of calls by visual inspection of all electropherograms. Power calculations using the software package Quanto [19] demonstrated that we had 90% power to detect a variant with a MAF of 1% and an OR of 2.5 for α = 0.05.

The background allele frequency of variants identified in this study was established in a UK control population using custom TaqMan assays (primer sequences included in supplementary table S1) on the ABI 7900HT platform (n = 350 for the 3 intronic SNPS and n = 1050 for the exonic variant identified). Genotype quality was assessed by evaluating the genotyping success rate (greater than 98% for all variants) and assessing whether there was any departure from Hardy-Weinberg Equilibrium (none was detected).

## Results

We identified a total of 4 variants (three intronic and one exonic) in resequencing the terminal 3 exons of *HNF1A* in 591 individuals (Table 2). However no novel coding variants (low frequency or otherwise) was identified. We subsequently genotyped a subset of the British Birth Cohort of 1958 (n = 350 for the intronic variants identified and n = 1050 for the exonic variant identified) as noted above, to establish the MAF of the variants identified in a UK population (Table 2). The only coding variant identified (T515T, rs61953349) is a previously reported synonymous variant with a MAF of 14.6% in our cases and of 18.3% in control individuals (p = 0.056, using an exact implementation of the Cochran Armitage trend test). For the 3 intronic variants, no statistically significant differences in genotype frequencies were noted between the cases and controls (calculated using an exact implementation of the Cochran Armitage trend test (Table 2).

**Table 2:** Variants identified in exons 8–10 of *HNF1A*.

Coding region	Approved cDNA level description	Description used in MODY literature	rs number	MAF current study (%)	MAF in population controls (%)
Intron 8	c.1623+29C>T	IVS8+29C>T	rs1169304	20.1	*17.9
Intron 9	c.1769−24T>C	IVS9−24T>C	rs735396	33.4	*34.3
Intron 9	c.1768+44C>T	IVS9+44C>T	N/A	4.1	*4.3
Exon 8	c.1545G>A	T515T	rs61953349	14.6	**18.3

N/A; not available.

* Based on screening 350 population controls (part of the UK 1958 Birth Cohort).

** Based on screening 1050 population controls (part of the UK 1958 Birth Cohort).

## Discussion

In a recent study of MODY families showing classical Mendelian segregation, individuals with mutations in exons 8–10 of *HNF1A* were noted to have been diagnosed as late as 38 years, and the median age of diagnosis was ∼27 years [3]. It was this observation that led us to test the hypothesis that coding variants within the same exons might be playing a role in the pathogenesis of multifactorial T2D. Given that genome-wide association studies have already surveyed common variation[17], [20]–[23], our particular focus was on variants of relatively low frequency (in the 0.5–5% range). If variants in this part of the allele frequency spectrum have effect sizes greater than those so far observed for common variants, they may be responsible for a substantial proportion of the genetic variance of T2D.

In our study population, 60 (10%) individuals were diagnosed at 38.5 years or younger and the median age of diagnosis was 55 (compared to an average age of diagnosis in the UK of 59.6 years [based on 15653 T2D patients from Tayside Scotland. R. McAlpine & AD Morris, personal communication]). Almost all cases had close relatives with T2D, a feature which is likely to reflect enrichment for medium penetrance variants.

Despite these measures, we failed to detect any novel coding variants in the terminal exons of *HNF1A* and can be confident that there are no such variants with case allele frequencies exceeding 1.0%. Of course, we cannot exclude the possibility that additional low-frequency susceptibility variants will be found in other regions of this gene, nor in other genes known to be causal for monogenic forms of diabetes. Recent advances in high throughput “next-generation” resequencing technologies [24] now make it feasible to deep-resequence multiple genes in large numbers of subjects in a cost effective manner, and should enable these broader hypotheses to be tested.

## Supporting Information

Table S1 Assay by design sequences for genotyping of variants detected in exons 8–10 of HNF1A
(0.03 MB DOC)
