# Supplementary material for: Low Frequency Variants in the Exons Only Encoding Isoform A of HNF1A Do Not Contribute to Susceptibility to Type 2 Diabetes
Source: PLoS One. 2009 Aug 12;4(8):e6615. doi: 10.1371/journal.pone.0006615 (PMC2720540; doi:10.1371/journal.pone.0006615)
Supplement: Table S1 — Assay by design sequences for genotyping of variants detected in exons 8–10 of HNF1A (0.03 MB DOC) [file pone.0006615.s001.doc]

**Supplementary Table S1.**

***Assay by design sequences for genotyping of variants detected in exons 8-10 of*** HNF1A

| **Approved cDNA level description** | **Forward Primer Seq.** | **Reverse Primer Seq.** | **Reporter 1 Sequence**  **(Dye label VIC)** | **Reporter 2 Sequence**  **(Dye label FAM)** |
| --- | --- | --- | --- | --- |
| c.1623+29C>T | CCCACCAAGCAGGTAAGGT | CAGAGCAGCCTCCTGAGC | CCCTCCCT**T**GGCCTGT | CCTCCCT**C**GGCCTGT |
| c.1769-24T>C | GGTGTGGGTGCCTGGT | GAGGACACTGCAGAGGCAAA | CAAGGCTGCT**A**GCCAC | AAGGCTGCT**G**GCCAC |
| c.1768+44C>T | CCACCCCCTCCCTTACTGT | AGTGACGGACAGCAACAGAA | CCCTGCCC**T**CTTCCAT | CCTGCCC**C**CTTCCAT |
| c.1545G>A | CGAGGTGGCCCAGTACAC | GTGTCGGTGATGAGCATAGTCT | CCACAC**A**GGCCTGC | CACAC**G**GGCCTGC |
